# In-hospital mortality of patients with acute coronary syndrome (ACS) after implementation of national health insurance (NHI) in Indonesia

**DOI:** 10.1186/s12913-024-10637-5

**Published:** 2024-03-05

**Authors:** Nurul Qalby, Dian S. Arsyad, Andriany Qanitha, Maarten J. Cramer, Yolande Appelman, Dara R. Pabittei, Pieter A. Doevendans, Idar Mappangara, Akhtar Fajar Muzakkir

**Affiliations:** 1 Department of Cardiology, University Medical Center Utrecht, Utrecht University, Utrecht, The Netherlands; 2 Department of Public Health, Faculty of Medicine, Hasanuddin University, Makassar, Indonesia; 3 Department of Epidemiology, Faculty of Public Health, Hasanuddin University, Makassar, Indonesia; 4 Department of Physiology, Faculty of Medicine, Hasanuddin University, Makassar, Indonesia; 5 Department of Cardiology, Cardiovascular Sciences, Amsterdam UMC Location VUMC, Amsterdam, the Netherlands; 6 Department of Cardiothoracic Surgery, AMC Heart Center, Amsterdam UMC Location AMC, Amsterdam, The Netherlands; 7 Netherlands Heart Institute, Utrecht, The Netherlands; 8 Central Military Hospital, Utrecht, The Netherlands; 9 Department of Cardiology and Vascular Medicine, Faculty of Medicine, Hasanuddin University, Makassar, Indonesia

**Keywords:** National health insurance, Acute coronary syndrome, In-hospital mortality, Cardiovascular healthcare

## Abstract

**Background:**

The National Health Insurance (NHI) was implemented in Indonesia in 2014, and cardiovascular diseases are one of the diseases that have overburdened the healthcare system. However, data concerning the relationship between NHI and cardiovascular healthcare in Indonesia are scarce. We aimed to describe changes in cardiovascular healthcare after the implementation of the NHI while determining whether the implementation of the NHI is related to the in-hospital mortality of patients with acute coronary syndrome (ACS).

**Methods:**

This is a retrospective comparative study of two cohorts in which we compared the data of 364 patients with ACS from 2013 to 2014 (Cohort 1), before and early after NHI implementation, with those of 1142 patients with ACS from 2018 to 2020 (Cohort 2), four years after NHI initiation, at a tertiary cardiac center in Makassar, Indonesia. We analyzed the differences between both cohorts using chi-square test and Mann-Whitney U test. To determine the association between NHI and in-hospital mortality, we conducted multivariable logistic regression analysis.

**Results:**

We observed an increase in NHI users (20.1% to 95.6%, *p* < 0.001) accompanied by a more than threefold increase in patients with ACS admitted to the hospital in Cohort 2 (from 364 to 1142, *p* < 0.001). More patients with ACS received invasive treatment in Cohort 2, with both thrombolysis and percutaneous coronary intervention (PCI) rates increasing more than twofold (9.2% to 19.2%; *p* < 0.001). There was a 50.8% decrease in overall in-hospital mortality between Cohort 1 and Cohort 2 (*p* < 0.001).

**Conclusions:**

This study indicated the potential beneficial effect of universal health coverage (UHC) in improving cardiovascular healthcare by providing more accessible treatment. It can provide evidence to urge the Indonesian government and other low- and middle-income nations dealing with cardiovascular health challenges to adopt and prioritize UHC.

**Supplementary Information:**

The online version contains supplementary material available at 10.1186/s12913-024-10637-5.

## Background

Universal health coverage (UHC) to ensure equal accessibility to healthcare is accepted worldwide as one of the fundamental factors needed to achieve sustainable development goals, as documented, and approved by many countries under the United Nations [1]. Equal accessibility is translated as the same level of effort required for each person to obtain the needed treatment. Ensuring healthcare accessibility in low- and middle-income nations remains a challenge, with various determinants at play, including UHC funding.

In Indonesia, the National Health Insurance (NHI) system, also known as the Jaminan Kesehatan Nasional (JKN), was started on 1 January 2014. Before that, there were many different health insurance schemes in Indonesia, with different types of coverage and requirements. The latest health insurance system implemented cross-subsidization funding, in which the contribution for poor people is paid by the central government, while workers in private companies and entrepreneurs pay their own contribution. The implementation of this system is done gradually with the target to have all Indonesian citizens covered by the NHI by the end of 2024 [2, 3].

Since the initial implementation of the NHI, noncommunicable diseases (NCDs) have been a concern of the government because of the large expenditure for the treatment of these diseases. Even though communicable infectious diseases have been known for decades to represent the predominant diseases in low- and middle-income countries, the prevalence of NCDs has also increased in relation to higher income per capita and changes in lifestyle risk factors, especially for middle-income citizens [4–7]. The top three causes of death in Indonesia from 2015 to 2019 were stroke, ischemic heart disease, and diabetes mellitus (DM) [8]. This shows the alarming position of NCDs in Indonesia today and in the near future.

According to data released by the Health Financing and Insurance Center, Indonesian Ministry of Health, 73.3% of public healthcare expenditures were spent on curative treatment, while only 9.6% funded patient education and prevention [9]. Data from Badan Penyelenggara Jaminan Kesehatan (BPJS), an institution that is responsible for the implementation of the NHI, show that cardiovascular diseases are the leading catastrophic condition with the highest burden of cost and account for the largest proportion of the funding expenditure since the start of the NHI [10]. 

Acute coronary syndrome (ACS) constituted the highest proportion of cardiovascular disease in Indonesia. Bagaswoto, et al., observed in SCIENCE (Sardjito Cardiovascular Intensive Care), that from 595 patients coming to the intensive care unit around January to November 2017, 69.9% were diagnosed with ACS and had an in-hospital mortality rate of 9.2% [11]. In a separate research conducted by Qanitha et al., it was revealed that approximately 76% of individuals within the coronary artery disease (CAD) population were afflicted by ACS during their study period spanning from February 2013 to December 2014 [12]. Furthermore, data sourced from the Center for Disease Control and Prevention (CDC) indicates that ACS and stroke are responsible for a substantial 80% of all cardiovascular-related fatalities in Indonesia [13]. 

Numerous studies have explored the impact of UHC on health outcomes, including mortality and life expectancy. A 2017 review found that insured individuals had mortality odds ratios ranging from 0.71 to 0.97 when compared to the uninsured [14]. The Organisation for Economic Cooperation and Development (OECD) reported positive effects of UHC on mortality and life expectancy in various age groups [15]. Nonetheless, some studies show no increased cardiovascular mortality risk for those insured through public insurance compared to the uninsured [16]. The effectiveness of UHC can vary between countries due to a multitude of factors, including political, socioeconomic, structural, healthcare system-related elements, patient characteristics, and regional disparities [17–19]. The implementation of NHI in Indonesia may present unique features and challenges, potentially affecting its impact on in-hospital mortality among patients with ACS in the Indonesian context.

With enormous funding of NHI spent on the treatment of cardiovascular diseases, such as ACS, to observe changes in the management and outcome after NHI implementation is expected. Previous studies showed the lack of UHC related to the obstacles and delays in treatment for ACS. Furthermore, differences in insurance type seem to cause disparities in the outcome of patients with ACS [20–24]. However, there remains a lack of available evidence regarding sex-based differences in this context [25]. To our knowledge, such study to examine post-NHI implementation’s outcome in patients with ACS has never been done in Indonesia. Thus, in this study, we aim to provide a description of the management of ACS before and after the implementation of the NHI and to determine whether the implementation of the NHI was related to the outcome of in-hospital mortality of patients with ACS.

## Methods

### Study population

This is a retrospective comparison study which included patients from two cohorts with a total of 1506 patients were included in this study, with 364 patients enrolled from Cohort 1 and 1142 patients from Cohort 2. Patients who had been included in Cohort 1 were excluded from Cohort 2. Included were patients who came to the Makassar Cardiac Center of Wahidin Sudirohusodo Hospital, Makassar, Indonesia, with symptoms similar to those of ACS and the final diagnosis at discharge were ACS. The cardiac center is the referral hospital for Sulawesi and eastern Indonesia and mainly admits patients from Makassar and other regions in South Sulawesi. Patient was excluded from this study if he/she died immediately in the emergency department or had an unclear diagnosis. The number of patients included in both cohorts reflected the population of patients with ACS admitted to this hospital from different periods. All study protocols were approved by the Ethics Committee and Institutional Review Board of the Faculty of Medicine, Hasanuddin University Makassar, Indonesia, and all methods were carried out in accordance with relevant guidelines and regulations.

#### Cohort 1

Data from Cohort 1 were derived from a prospective cohort study performed by Qanitha et al. [12], in which data were collected from February 2013 to December 2014 (23 months). The data comprised 364 patients who were diagnosed with unstable angina (UA), non-ST elevated myocardial infarction (NSTEMI), and ST-elevated myocardial infarction (STEMI). The diagnosis was made by the cardiologist within 24 h of hospital presentation based on clinical presentation, electrocardiography, and changes in cardiac enzyme levels if available. The data were collected by trained research assistants from medical records and interviews. In-hospital mortality and treatment data were collected during hospitalization. All eligible patients provided informed consent before the initial interview for using their data.

#### Cohort 2

Cohort 2 utilized data from 1142 patients enrolled in the Makassar ACS Registry, initiated in June 2018 to gather data on ACS admissions. The study retrieved registry data until January 2020 (20 months). This registry is part of the national ACS registry, which was initiated by the Indonesian Cardiologist Association to assess data on the management of ACS in network hospitals. The diagnoses of UA, NSTEMI, and STEMI were made by cardiologists in the Makassar Cardiac Center within 24 h after patients’ admission based on the same criteria as those in Cohort 1. The data were collected from hospital medical records into an electronic case report form (e-CRF) by a research assistant. In-hospital management and mortality data were also updated into the e-CRF at the end of the hospitalization. Patients included in this study gave their consent. (Additional detailed descriptions of methods, such the definitions of measured variables, are available in a supplementary document (see Supplementary material 1).

#### Patient involvement

Our study did not involve patients or members of the public in the concept or analysis of the research. We intend to disseminate the results of this study to members of the public and BPJS, the organization that arranges the NHI.

### Health insurance system

The Jaminan Kesehatan Nasional (JKN) is the primary health insurance system in Indonesia, introduced on January 1, 2014, following various transformations of the country’s insurance schemes. Initially, it didn’t encompass all citizens immediately, but covered groups like civil servants, military personnel, retirees, and vulnerable populations under previous government health insurance. Over time, it gradually integrated other programs, allowing those without insurance or with private coverage to join by paying contributions. While mandatory, by early 2021, roughly 20% of the population had not enrolled in the JKN program [2, 3]. 

### Statistical analysis

We aimed to ensure data compatibility between Cohort 1 and Cohort 2 by harmonizing variable definitions and units. We initially compared variable definitions in both cohorts to identify and rectify any substantial differences. The unit of each variable was established using Cohort 1 as a reference, and subsequently refined for Cohort 2. Finally, we merged the data from both cohorts into a single dataset to achieve compatibility. Then the data were tested with the Shapiro–Wilk test for normality. Continuous variables are presented as the mean ± SD, and skewed data are presented as the median (Q1-Q3). Baseline characteristics, diagnosis, treatment, and outcome were divided into Cohort 1 and Cohort 2 groups. To identify variable differences between Cohort 1 and Cohort 2, comparison tests were performed. For categorical data, we used the chi-square test. For continuous data, the Mann–Whitney U test. Following that, we conducted bivariable logistic regression analysis for each variable to identify any individual associations with in-hospital mortality. An independent association was considered when the bivariable analysis of dependent variables (age, sex, NHI, education level, active smoker, obesity, diabetes, hypertension, dyslipidemia, previous stroke, previous myocardial infarction, previous CHF, previous percutaneous coronary intervention (PCI), previous coronary artery bypass graft (CABG), acute heart failure, cardiogenic shock, stroke, STEMI, NSTEMI, UA, ASA, clopidogrel, statin, anticoagulant, thrombolysis, PCI, time from symptoms onset to admission, time from admission to intervention, duration in hospital) and in-hospital mortality as independent variables yielded p-values below 0.25. Following this, a multivariable logistic regression analysis was undertaken to calculate the Relative Risk (RR) for significant variables (age, sex, NHI, active smoker, obesity, diabetes, hypertension, dyslipidemia, acute heart failure, cardiogenic shock, stroke, PCI, and time from symptoms onset to admission). This step aimed to control for confounding variables, providing a more precise evaluation of each variable’s independent impact on the outcome while identifying the most influential predictors. In our analysis, we evaluated multicollinearity among the variables included in the analysis through the Variance Influence Factor (VIF) test, aiming to enhance the model’s robustness. The selection of these variables aligned with the study’s objectives and theoretical framework. Importantly, all incorporated variables exhibited VIF values below 10. Finally, a forest plot was generated to visualize the results. An RR greater than 1 indicates an increased risk, while an RR less than 1 suggests a decreased risk. The result was statistically significant if a two-sided p-value was less than 0.05. We used IBM SPSS Statistics V.27.0 (IBM-SPSS Inc., Chicago, USA) to perform all the statistical analyses, together with GraphPad Prism (GraphPad Software, Inc., CA, US) and Microsoft® Excel® for Microsoft 365 MSO Version 2203 (Microsoft Corporation, Redmond, WA, USA) for visualization.

## Results

### Baseline characteristics

**Table 1:** Comparison of baseline characteristics between Cohort 1 and Cohort 2

Baseline characteristics	Cohort 1 (n = 364)	Cohort 2 (n = 1142)	p value
**Male sex**	260 (71.4)	847 (74.2)	0.302
**Age (years)**	57.46 ± 11.24	58.16 ± 10.82	0.290
**Healthcare Insurance**			
- No insurance	30 (8.2)	48 (4.2)	< 0.001*
- NHI	73 (20.1)	1092 (95.6)	< 0.001*
- Government funded insurance	199 (54.7)	0 (0)	< 0.001*
- Employee/regional government/private insurance	62 (17.0)	1 (0.1)	< 0.001*
**Education level**			
- Low education (illiterate– junior high)	119 (32.7)	383 (33.5)	0.201
- High education (senior high– doctorate)	244 (67.0)	759 (66.5)	0.790
**Smoking**			
**-** Current smoker	103 (28.3)	494 (43.3)	< 0.001*
- Former smoker	118 (32.4)	220 (19.3)	< 0.001*
**Obesity (Asian classification, BMI ≥ 25 kg/m** ^**2**^ **)**	119 (32.7)	336 (29.4)	0.237
**Medical history**			
- Diabetes	112 (30.8)	338 (29.6)	0.086
- Hypertension	275 (75.5)	1004 (87.9)	< 0.001*
- Dyslipidemia	280 (76.9)	706 (61.8)	0.001*
- Previous stroke	27 (7.4)	51 (4.9)	0.027*
- Previous myocardial infarction	126 (34.6)	167 (14.6)	< 0.001*
- Previous CHF	92 (25.3)	139 (12.2)	< 0.001*
- Previous PCI	14 (3.8)	95 (8.3)	0.004*
- Previous CABG	0 (0)	9 (0.8)	0.089
**Concomitant condition**			
- Acute heart failure (≥ Killip 2)	150 (41.2)	349 (30.6)	< 0.001*
- Cardiogenic shock	17 (4.7)	72 (6.3)	0.194
- Stroke	18 (4.9)	23 (2.0)	0.004

Values are presented as n (%) or mean ± SD for continuous variables. Comparisons were analyzed using an independent t test for continuous variables and Pearson’s chi-square test for categorical variables. * indicates a statistically significant difference (*p* < 0.05). Cohort 1 = February 2013 - December 2014 Makassar ACS Cohort study, Cohort 2 = June 2018 - January 2020 Makassar ACS Registry. NHI = National Health Insurance. BMI = body mass index. CHF = congestive heart failure. PCI = percutaneous coronary intervention. CABG = coronary artery bypass graft

Table 1 shows the baseline characteristics of both cohorts. There was a substantial increase in the number of patients admitted in Cohort 2 compared to Cohort 1; in Cohort 1 with 364 patients with ACS were recorded, while in Cohort 2, the number of patients admitted with ACS was increased more than 3-fold to 1142 patients. No difference was found in age with mean 57.81 years old in both cohorts and the majority of the population were male participants. However, females in our population tended to be older than males in both cohorts (Cohort 1: females 62.1 ± 10.2 years old, males: 55.6 ± 11.1 years old; Cohort 2: females 61.0 ± 10.1 years old, males : 57.2 ± 10.9 years old). Also, no discrepancy was seen in education level between the participants in both cohorts. However, there was significant increase from 20.1 to 95.6% (*p* < 0.001) in the percentage of NHI users in Cohort 2 compared to Cohort 1. Furthermore, there was a considerable increase in the number of both men and women with hypertension in cohort 2 compared to cohort 1. Specifically, the increase in hypertension was slightly higher in men (74.2% to 87.7%, *p* < 0.001) than in women (78.9% to 88.9%, *p* = 0.011) respectively.

In addition, in Cohort 2 compared to Cohort 1, there was an increase in the percentage of people who were active smokers, from 28.3 to 43.4% overall (*p* < 0.001). The number of women who smoked increased from 0.0 to 4.41% (*p* = 0.030), while for men, the increase was approximately 1.5-fold from 39.6 to 59.8% (*p* < 0.001). On the other hand, variables such as dyslipidemia, previous stroke, previous myocardial infarction, previous congestive heart failure, and a history of previous PCI dropped in Cohort 2 compared to Cohort 1. Diabetes mellitus, one of the classic risk factors for ACS, was shown to be reduced in cohort 2; nevertheless, there was no difference found between the groups of the cohort (30.8% to 29.6%, *p* = 0.086). Regarding gender differences, women tended to have a higher prevalence of diabetes compared to men in both cohorts (Cohort 1: women 40.4%, men 26.9%, *p* = 0.012; Cohort 2 : women 41.7%, men 25.4%, p = < 0.001).

Concomitant conditions, i.e., acute heart failure and stroke, also seemed to be significantly decreased in Cohort 2 after NHI implementation (41.2% to 30.6%, *p* < 0.001; 4.9% to 2.0%, *p* = 0.004). On the other hand, the number of patients with concomitant cardiogenic shock was observed to increase by approximately 25% in Cohort 2 compared to Cohort 1. In Cohort 2, women were also more likely to present with HF or concomitant stroke compared to men (HF, *p* = 0.009; concomitant stroke, *p* = 0.047).

### Diagnosis and treatment

**Table 2:** Comparison of diagnosis, treatment, and outcome variables between Cohort 1 and Cohort 2

Variables	Cohort 1 (N = 364)	Cohort 2 (N = 1142)	p value
	n (%)	n (%)	
**Diagnosis**			
UA	68 (18.7)	157 (13.7)	0.021*
NSTEMI	85 (23.4)	389 (34.1)	< 0.001*
STEMI	211 (60.0)	594 (52.0)	0.047*
**Treatment and outcome**			
***Intrahospital pharmacotherapy***			
ASA/aspirin	320 (87.9)	1118 (97.9)	< 0.001*
Clopidogrel	308 (84.6)	1086 (95.1)	< 0.001*
Statin	271 (74.5)	1112 (97.4)	< 0.001*
Anticoagulant	168 (46.2)	1047 (91.7)	< 0.001*
***Invasive treatment***			
Thrombolysis	13 (3.6)	163 (14.3)	< 0.001*
PCI - Primary PCI - Rescue PCI	34 (9.2) 10 (2.7) 2 (0.6)	220 (19.2) 72 (6.3) 16 (1.4)	< 0.001* 0.009* 0.196
Time from onset to admission (hours) ^β^	24 (9–48)	13 (6–33)	< 0.001*
Time from admission to intervention (hours) ^β^	120 (8–168)	19.58 (13.2–81.4)	< 0.001*
Duration in hospital (days) ^β^	7 (5–10)	6 (5–7)	0.002*
**In-hospital mortality**	46 (12.6)	66 (5.8)	< 0.001*

Values are presented in n (%) and analyzed using Pearson’s chi-square test. ^β^Values presented as the median (Q1-Q3) were analyzed using the Mann–Whitney U test. * p values < 0.05 indicate statistically significant differences. Cohort 1 = February 2013 - December 2014 Makassar ACS Cohort study, Cohort 2 = June 2018 - January 2020 Makassar ACS Registry. UA = unstable angina. NSTEMI = non-ST-segment elevation myocardial infarction, STEMI = ST-segment elevation myocardial infarction. ASA = acetyl salicylic acid. PCI = percutaneous coronary intervention

Table 2 shows that there were fewer patients diagnosed with UA in Cohort 2, yet the numbers of patients diagnosed with NSTEMI and STEMI significantly increased (NSTEMI, from 17.8 to 34.1%, *p* < 0.001; STEMI, from 44.2 to 52.0%, *p* = 0.047). Most of the variables in Table 2 exhibited substantially different proportions between Cohort 1 and Cohort 2 (*p* < 0.05). For in-hospital drug therapy, ASA, clopidogrel, or anticoagulants were prescribed more frequently in Cohort 2 than in Cohort 1 (*p* < 0.001). The rise in drug utilization was in line with the increase in NHI users among patients with ACS. In both Cohort 1 and Cohort 2, anticoagulant therapy was administered as an adjuvant for patients with ACS after ASA and clopidogrel.

Furthermore, per treatment procedure, the number of PCI procedures performed in Cohort 2 doubled in comparison to that in Cohort 1 (*p* < 0.001). This was also in accordance with the increased proportion of thrombolysis/fibrinolysis treatment (from 3.6 to 14.3%, *p* < 0.001). More specifically, men tended to undergo PCI more often than women in both Cohort 1 and 2, while for thrombolysis, women were more frequently treated than men in Cohort 2. The rates of PCI treatment in men increased from 10.4 to 20.4% (*p* < 0.001), and the same trend was seen in women, where PCI rates rose from 3.8 to 15.6% (*p* < 0.001). The rate of primary PCI increased more than twofold from Cohort 1 to Cohort 2 (2.7% to 6.3%, *p* = 0.009). The overall CAG rates were 23.1% in Cohort 1 and increased to 50.3% in Cohort 2 (*p* < 0.001). While for patients with NSTEMI, there was also a significant increase in CAG rates in Cohort 2 compared to Cohort 1 (17.7% to 31.8%, *p* < 0.001).

Additionally, the duration between the onset of symptoms to admission in both cohorts differed substantially from a median of 24 h in 2013–2014 to 13 h in 2018–2020. This difference was extended even more for the delay between admission to the hospital and intervention, where the median time in Cohort 1 was 120 h, while that in Cohort 2 was 19.58 h. Furthermore, fewer people stayed in the hospital for more than 8 days after admission in Cohort 2 compared to those in Cohort 1 (decreased from 34.6 to 13.7%, respectively *p* < 0.001). Finally, as represented in Table 2, the percentage of in-hospital deaths decreased significantly by almost 50%, from 11.8% in Cohort 1 to 5.8% in Cohort 2 (*p* < 0.001).

### Outcomes and predictors

**Fig. 1:**
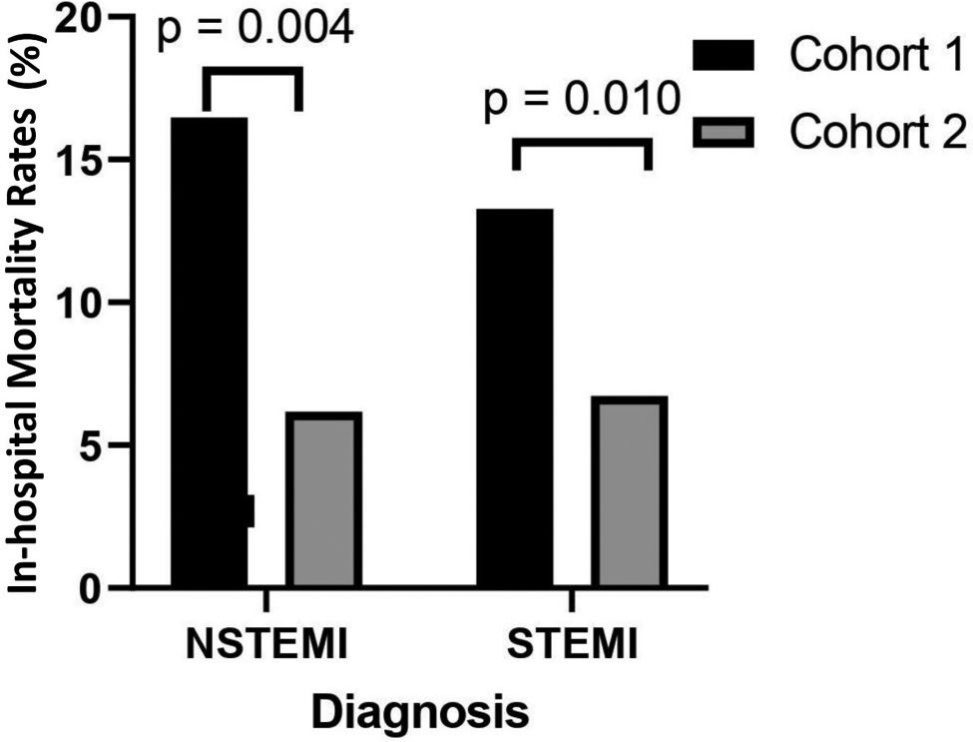
In-hospital mortality rates by ACS diagnosis in Cohort 1 and Cohort 2. Y-axis represents in-hospital mortality rates as a percentage for NSTEMI and STEMI. The black bar indicates the rates in Cohort 1, whereas the gray bar indicates the rates in Cohort 2. On the upper part of the bars for each diagnosis, there is a p value indicating the significance of the difference between Cohort 1 and Cohort 2, and a p value < 0.05 indicates statistical significance. Cohort 1 = 2013–2014 Makassar ACS Cohort study, Cohort 2 = 2018–2020 Makassar ACS Registry, NSTEMI = non-ST-segment elevation myocardial infarction, STEMI = ST-segment elevation myocardial infarction

**Fig. 2:**
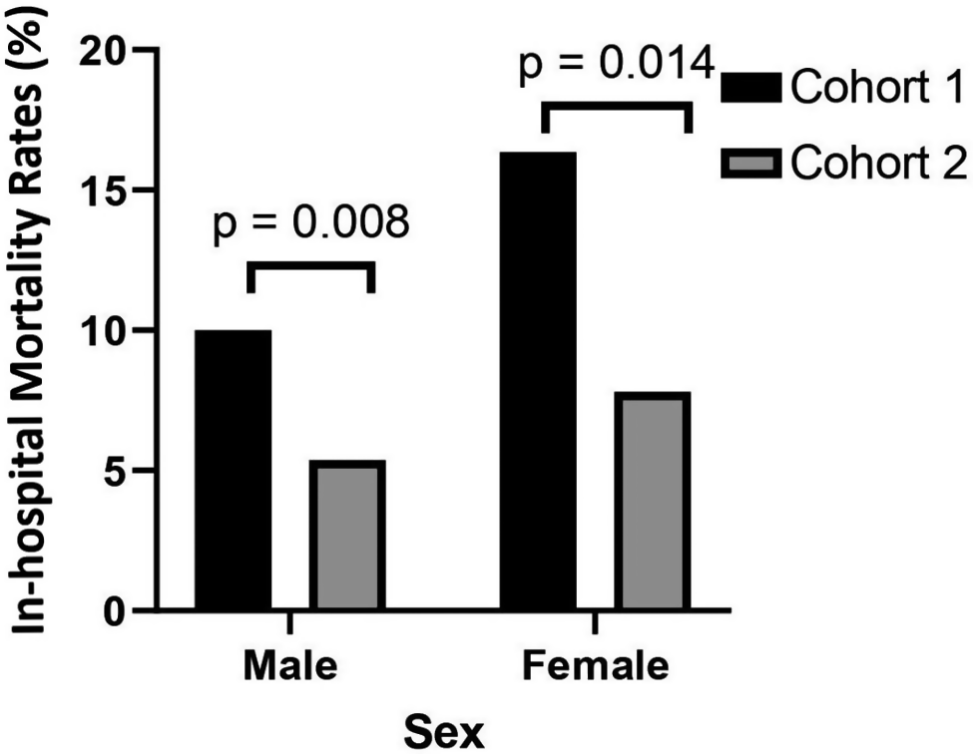
In-hospital mortality rates by sex in Cohort 1 and Cohort 2. The vertical line represents in-hospital mortality rates as a percentage for males and females. The black bar indicates the rates in Cohort 1, whereas the gray bar indicates the rates in Cohort 2. On the upper part of the bars on each diagnosis, there is a p value indicating the significance of the difference between Cohort 1 and Cohort 2, and a p value < 0.05 indicates statistical significance. Cohort 1 = 2013–2014 Makassar ACS Cohort study, Cohort 2 = 2018–2020 Makassar ACS Registry, NSTEMI = non-ST-segment elevation myocardial infarction, STEMI = ST-segment elevation myocardial infarction

**Fig. 3:**
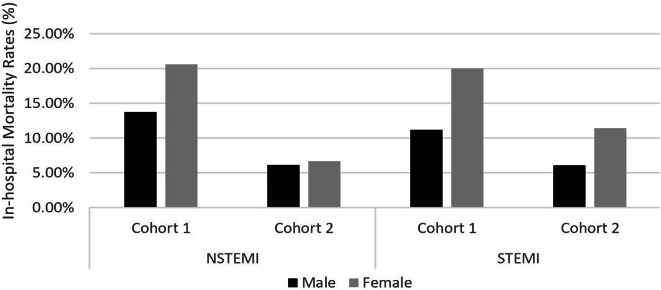
In-hospital mortality rates by ACS diagnosis and sex in Cohort 1 and Cohort 2. The vertical line represents in-hospital mortality rates as a percentage for NSTEMI and STEMI. The black bar indicates the rates in males, whereas the gray bar indicates the rates in females. Cohort 1 and Cohort 2 are side by side in each group of patients with NSTEMI and STEMI. Cohort 1 = 2013–2014 Makassar ACS Cohort study, Cohort 2 = 2018–2020 Makassar ACS Registry, NSTEMI = non-ST-segment elevation myocardial infarction, STEMI = ST-segment elevation myocardial infarction. The data in this figure were presented in their raw, unadjusted form, and no statistical analysis was conducted on them

In accordance with Table 2; Fig. 1 shows the rates of in-hospital mortality, which decreased substantially in Cohort 2 compared to Cohort 1. Specifically, for patients diagnosed with STEMI and NSTEMI, the rates were reduced by half compared to those in Cohort 1 (*p* = 0.010 (STEMI), *p* = 0.004 (NSTEMI)). On the other hand, there was only a slight decrease observed in the number of patients with UA in relation to in-hospital mortality rates. While observing a decreasing trend in in-hospital mortality rates following the implementation of the NHI, it’s noteworthy that mortality rates decreased significantly across cohorts for both sexes (*p* = 0.014 for women, *p* = 0.008 for men) as depicted in Fig. 2. This overall decline persists consistently across sexes, although a slight but significant sex-based difference in mortality rates remains evident. Once age was taken into account, the gender-related variations in mortality rates for both STEMI and NSTEMI diagnoses lost significance, as women tended to be 5–7 years older than men at presentation (*p* = 0.146). Also, as shown in Fig. 3, women in Cohort 2 who were diagnosed with STEMI had higher mortality rates than men with the same diagnosis. In the NSTEMI group, the in-hospital mortality between men and women was comparable. Subsequently, age and sex were also adjusted for both STEMI (*p* = 0.075) and NSTEMI (*p* = 0.160) and the differences observed previously were no longer seen.

**Fig. 4:**
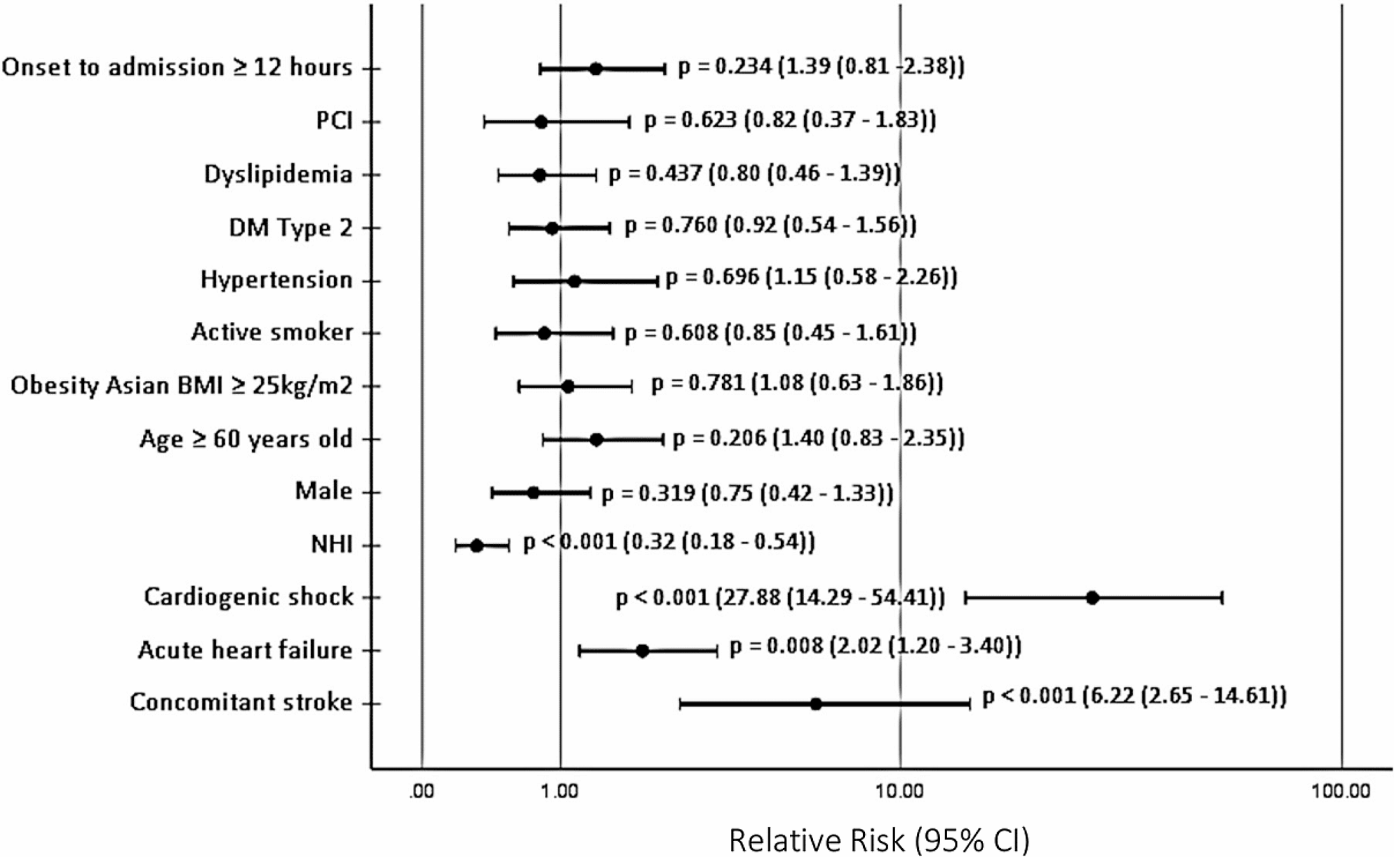
Multivariable logistic regression analysis of in-hospital mortality outcome. Forest plot presenting multivariable relative risks for in-hospital mortality. The horizontal line incorporates the range of the lower and upper 95% confidence intervals, and the dot represents the relative risk. PCI = percutaneous coronary intervention. DM Type 2 = diabetes mellitus type 2. BMI = body mass index. NHI = National Health Insurance

As shown in Fig. 4, the NHI, along with concomitant conditions (acute heart failure, concomitant stroke, and cardiogenic shock), was found to be an important factor that influenced in-hospital mortality in this study. The implementation of the NHI was found to significantly reduce in-hospital mortality (RR 0.32 (95% CI 0.18–0.54), *p* < 0.001) after adjustment for sex, age, hypertension, diabetes mellitus type 2, dyslipidemia, obesity, active smoking, PCI, and time from onset to admission of more than 12 h, while patients with ACS who had cardiogenic shock, acute heart failure, or concomitant stroke had at least twice the chance of in-hospital death (cardiogenic shock RR 27.88 (95% CI 14.29–54.41), *p* < 0.001; acute heart failure RR 2.02 (95% CI 1.20–3.40), *p* = 0.008; concomitant stroke RR 6.22 (95% CI 2.65–14.61), *p* < 0.001). Those who had NHI coverage had a 68% lower in-hospital mortality rate (*p* < 0.001), while patients who had concomitant conditions of acute heart failure, concomitant stroke, and cardiogenic shock had approximately 2-fold, 6-fold, and 30-fold higher in-hospital mortality rates, respectively, than those who did not.

## Discussion

To our knowledge, this is the first study to assess ACS management in relation to the implementation of the NHI in Indonesia. We used two datasets from different periods, i.e. one including data prior and the other including data post implementation of NHI and included patients with ACS who came to our cardiac center. In-hospital mortality was selected as the outcome to observe the effect of the NHI in both cohorts. The present study showed an increase in NHI utilization and lower in-hospital mortality among patients with ACS after the start of the NHI program. This reduction was seen more prominently among patients with NSTEMI when comparing Cohort 1 to Cohort 2 than among patients with STEMI within the same cohorts. Both men and women experienced a decrease in in-hospital mortality. Furthermore, ACS treatment utilization as a proxy for healthcare access was observed to increase considerably after NHI implementation.

A decrease in overall in-hospital mortality was observed after the implementation of the NHI. Specifically, for NSTEMI and STEMI, the rates dropped by 50%. The decreasing number could be due to reductions in the duration from the onset of complaints to admission and the duration from hospital admission to PCI. Additionally, more patients received thrombolysis and underwent PCI; thus, the accessibility to the hospital and subsequent treatment after the implementation of the NHI might influence the reduction seen in this study.

Extended delays in Cohort 1, surpassing the recommended 120-minute guideline [26] for invasive interventions, result from various systemic factors, including high out-of-pocket expenses, limited healthcare benefits awareness, regional disparities in access, supply-side readiness challenges, and care quality issues. Delays at healthcare facilities may stem from medicine availability and inpatient care constraints, leading to financial strain for insurance beneficiaries. Social and cultural beliefs, insurance complexity, and perceived service quality limit prompt help-seeking, while elective surgery wait times are also extended, largely due to administrative and resource-related issues. Despite these systemic challenges, Cohort 2 exhibits significant improvements, although it still falls short of full guideline adherence [26]. Based on a review by Mol KA et al [27], the reduction in prehospital delays may influence the mortality of both patients with NSTEMI and STEMI, as the pathophysiology of NSTEMI and STEMI is comparable. In our study, NSTEMI mortality was found to decrease more than STEMI mortality. This could be explained by the fact that the number of primary PCI as treatment for STEMI was still low compared to CAG within 24 h, which was recommended for NSTEMI [28]. Despite a reduction in system delay (times from onset to admission and admission to reperfusion), the targeted maximum time of 90 min for system delay for STEMI has not yet been achieved.

When analyzed by sex, both men and women populations in this study showed a decreasing trend in in-hospital mortality. Furthermore, despite women constituting approximately one-fourth of the population compared to men, they exhibited higher mortality rates. Specifically, an increased in-hospital mortality rate was noted for women compared to men, particularly among patients with STEMI in Cohort 2. Although this difference in in-hospital mortality rate disappeared after adjusting for age in our study, it is worth noting that it aligns with the results from several studies, where women had higher rates of in-hospital mortality than men due to lower treatment rates in women, such as less double antiplatelet therapy or women receiving lower rates of PCI [24, 29–33]. Furthermore, women tend to experience more delays, both from onset to admission and admission to treatment, which could be caused by their dismissal of symptoms or the symptoms themselves that were not typical of ACS symptoms [29]. Besides that, women in our study tend to be older than men with around 5–7 years difference as women develop obstructive coronary artery disease after menopause as a result of losing the protective effect of estrogen. Women with diabetes are also have a higher risk of CHD compared to men [33, 34]. These factors could be the precipitators of women’s higher in-hospital mortality rate in our study. However, a study published in 2021 which categorized patients with STEMI into groups based on the presence of standard modifiable cardiovascular risk factors (SMuRFs) found that women without SMuRFs had higher rates of in-hospital mortality in their study, attributing the lower rates of evidence-based pharmacotherapy given to this group due to their lack of risk factors [35]. Thus, ACS in women should also be highlighted in both education and prevention programs in primary healthcare and curative in-hospital treatment, also for the secondary prevention program.

Since the recommendation of anticoagulants in European Society of Cardiology (ESC) guidelines for patients with STEMI and NSTEMI was published in 2015 [26, 36], the utilization of anticoagulants for those patients has doubled. Anticoagulant therapy was indicated for all patients with ACS, along with administration of double antiplatelet therapy. In our study, those who had contraindications to anticoagulants or were found to have no major stenosis after PCI did not receive anticoagulant therapy.

For revascularization therapy, thrombolysis was the most frequent therapy administered to patients with ACS compared to PCI in this study. The utilization of both therapies increased after the implementation of the NHI. From the type of thrombolytic agent, alteplase was more commonly accessible in Indonesia after it was added as recommended therapy covered by NHI. Regarding the rates, the proportion of the use of both therapies increased more than 100% after initiation of the NHI, indicating the influence of the NHI on therapy accessibility. However, there was a difference in rates of CAG and PCI between men and women, where women had lower CAG and PCI rates, which might also influence the outcome, especially for patients with STEMI leading to a higher mortality in women. This could indicate undertreatment of ACS in women in our study, and further study on the cause of the difference in CAG and PCI rates between men and women is needed. Expert consensus in 2020 also further elaborated that women more often have non-obstructed coronary artery disease and less often undergo CAG than men; thus, they may often receive fewer PCI procedure [37]. 

In Indonesia, districts have tiered hospitals labeled as types D, C, B, and A, each with a varying number of medical specialists available. Patients visit their district’s type D hospital and are then referred based on their condition’s severity and required treatment. Those near higher-tier hospitals needing emergency care can directly access them. Once stabilized, patients are discharged and guided for follow-up care at a suitable lower-tier hospital [38–43]. 

From Cohort 1 to Cohort 2, we concluded that more people who experienced the symptoms of ACS came to the Makassar Cardiac Center in Wahidin Sudirohusodo Hospital, which serves as a type A hospital. Compared to that in other regions in Indonesia, utilization of inpatient care in Sulawesi was already the highest based on a study by Mulyanto J [44], et al. from the 2013 Basic Health Research Survey, which was done before the implementation of the NHI. Nonetheless, inpatient care utilization seemed to have increased even more in Cohort 2 after the implementation of the NHI. Similar educational backgrounds in both cohorts may indicate that there are factors outside of education that influence the increase in the awareness of the patients to come earlier to the hospital or to access timely medical assistance. The increase in hypertension rates in our study may indicate more screening of hypertension outside of Java and Bali, even though it took a few more years from the start of the NHI, which could signify the spread of health equity in a broader area of Indonesia after the NHI was implemented.

In a broader context, the increase in hypertension as a risk factor for NCDs should also be an alarming indicator. From the baseline characteristics, the prevalence of hypertension was higher in women than men in both cohorts. This result is in line with those from the literature comparing hypertension and ACS between men and women, where a hypertension background was more often seen in women than in men [29, 31]. Thus, apart from better ACS awareness, primary care may also need to prepare for the strategic prevention and control of hypertension in the community.

Another major concern is the substantial increase in the number of active smokers. Smoking also contributes to an increased risk of cardiovascular diseases [45]. We can see that the number of active smokers increased in Cohort 2 in the era after the implementation of the NHI. The increased number of active smokers was seen for both men and women, but this increase was substantially higher for women. Based on a review performed by Appelman Y, et al. [34], in 2014, women who smoke might have a greater relative risk for coronary heart disease than men who smoke. In the past, women who smoke tended to start later in life with fewer cigarettes than men; however, women in the newer era tended to start smoking at the same age as men with the same number of cigarettes.

The significant increase in hypertensive patients and active smokers in Cohort 2 compared to Cohort 1 can be attributed to multifaceted factors, including urbanization, the aging of the population, changes in dietary patterns, a more sedentary lifestyle influenced by the proliferation of food delivery apps, and heightened social stress [46–49]. Hypertension has been a global concern with a substantial increase expected by 2017 ACC/AHA [50]. In Indonesia, the prevalence of hypertension among patients with coronary heart disease (CHD) varies across regions, ranging from 21 to 42%. According to a study, the prevalence is 51% among patients with ACS, making the prevalence of hypertensive patients in our data exceptionally high [51, 52]. Smoking prevalence among patients with ACS or CHD is also increasing worldwide. In China, the prevalence of active smokers among patients with ACS was 52.4%, while in selected European countries, the prevalence was 19.4% among patients with CHD in 1995, and in the United States, the prevalence was 32.4% in acute myocardial infarction patients in 2017 [53–55]. Our data shows that the prevalence of active smokers in our population is in line with the global trend, although it remains below that of China.

Before the era of the NHI, access to specific cardiac enzyme tests for ACS was limited, especially for those covered by regional government insurance. In instances where cardiac enzyme tests are financially inaccessible, our diagnostic approach hinges on thorough clinical assessments. Patients presenting symptoms are initially labeled as UA. Continuous clinical monitoring during hospitalization then determines the final UA diagnosis if patients remain stable without signs of myocardial damage or ongoing issues. Consequently, the higher proportion of patients with UA in Cohort 1 could likely be due to the unavailability of these tests for diagnosing NSTEMI. After the implementation of NHI, the number of patients diagnosed with UA decreased, while the number of patients diagnosed with NSTEMI increased. Additionally, the availability of serial high-sensitivity troponin test results to diagnose NSTEMI after the NHI could also explain the increase in NSTEMI diagnoses.

Compared to studies conducted in other countries focusing on UHC, our study has encountered distinct challenges. These include deficiencies in healthcare infrastructure, uneven resource allocation, readiness on the supply side, lower trust in public healthcare facilities, the impact of population aging, and disparities in healthcare accessibility. In contrast, Indonesia’s NHI program already encompasses a substantial portion of its population, while other countries struggle with expanding the program’s coverage. Additionally, variations exist in program design and financial allocation across countries, influenced by distinct policies and regulations within each nation [56, 57]. 

To advance cardiovascular healthcare in Indonesia, key recommendations from this study emerge. The NHI played a pivotal role, showing a significant 68% decrease in in-hospital mortality among covered patients. This emphasizes the need to expand healthcare access through NHI for further mortality reduction. Additionally, a focus on prevention is crucial, advocating healthy lifestyle promotion, improved screening, and increased public awareness of cardiovascular risks. Investing in primary care, especially in remote areas, is vital, necessitating better quality and availability of services and promoting healthier lifestyles. Improving healthcare infrastructure to reduce treatment delays, enhancing medical facility access, and upgrading equipment are also critical. Lastly, prioritizing research investments to understand prevention and treatment strategies is essential to fill knowledge gaps and innovate interventions. These recommendations outline a comprehensive plan for advancing cardiovascular prevention and control in Indonesia.

Our study has several limitations. The study included only patients from a hospital (Wahidin Sudirohusodo) in central Indonesia that serves as a national referral hospital for central and eastern Indonesia. However, this may not represent the situation in other regions of Indonesia, especially western Indonesia, since there are variations in health utilization, geographic factors, socioeconomic conditions, healthcare resources, cultural beliefs, and health seeking behaviors among different regions of Indonesia. Furthermore, Wahidin Sudirohusodo Hospital, a government hospital, may be preferable for patients with lower and middle-income backgrounds; thus, our study may have overlooked a minority of patients with higher incomes who may prefer private hospitals. Another limitation of our study is that it is a retrospective study; therefore, it is possible that our study does not capture all aspects of the outcome and might tend to have biases, specifically selection bias, such as variations in patients’ characteristics or differential attrition. There are also several changes associated with the 5-year gap that may have implications on the in-hospital mortality rate, for example infrastructure improvements, enhanced information accessibility, a growing number of cardiologists and resources, advanced technology adoption, rising income levels, diversified transportation options, expanded medication availability, increased health interventions, and the implementation of updated guidelines.; nevertheless, the change during these periods may be influenced by referral regulations, human resources, and facility enhancements provided by the NHI. Another limitation in this study arises from the unavailability of data from cohort 1, which constrained our capacity to evaluate the impact of a higher proportion of patients with STEMI and door-to-balloon time and door-to-wire crossing time on in-hospital mortality. Lastly, while the awareness of patients in both cohorts was not directly assessed in this study, it is noteworthy that after 2014, there has been a significant increase in internet connectivity and smartphone usage in Indonesia, which could have potentially influenced the earlier arrival of cohort 2 patients compared to cohort 1. This aspect may warrant consideration in future research for a more comprehensive understanding of its impact.

## Conclusions

After implementation of the NHI program, both men and women experienced enhanced accessibility to healthcare and utilization of revascularization therapy, which resulted in overall lower in-hospital mortality in our study. Our study underscores the importance of comprehensive policy changes in the field of cardiovascular prevention and control. To enhance healthcare in Indonesia, there is a pressing need for policies that address active smoking through smoking cessation programs and smoking bans, intensify awareness campaigns to promote early symptom recognition and the benefits of timely treatment, improve education regarding the referral system and health insurance, ensure equitable distribution of medicine and healthcare resources across regions, and implement enhanced monitoring, especially for high-risk populations, along with early screening programs. These policy adjustments can significantly contribute to the advancement of cardiovascular healthcare in the country. Thus, the implementation of the NHI as a program to achieve universal health coverage might be instrumental in improving cardiovascular healthcare in Indonesia. Further improvements in promotion and prevention in primary care are also needed to accomplish a further long-term reduction in the incidence of ACS.

### Electronic supplementary material

Below is the link to the electronic supplementary material

**Supplementary Material 1:** Methods - The definitions of variables measured in this study

## Data Availability

The dataset analyzed during this study are included in the published article are available from the corresponding author at nurulqalby@med.unhas.ac.id on reasonable request.

## Abbreviations

ACS: acute coronary syndrome
ASA: acetylsalicylic acid
ASKES: Asuransi Kesehatan (Indonesian term for health insurance)
BMI: body mass index
BPJS: Badan Penyelenggara Jaminan Sosial (Indonesian term for Social Security Organization that manage NHI)
CABG: coronary artery bypass grafting
CAD: coronary artery diseases
CAG: coronary angiography
CDC: Centers for Disease Control and Prevention
CHD: coronary heart diseases
CHF: chronic heart failure
DM: diabetes mellitus type 2
ESC: European Society of Cardiology
E-CRF: electronic case report form
HbA1c: hemoglobin A1c
HDL: high-density lipoprotein
JAMKESDA: Jaminan Kesehatan Daerah (Indonesian term for regional health insurance)
JAMKESMAS: Jaminan Kesehatan Masyarakat (Indonesian term for community health insurance)
JAMSOSTEK: Jaminan Sosial Ketenagakerjaan (Employment Social Security)
JKN: Jaminan Kesehatan Nasional (Indonesian term for NHI)
JNC VII: Joint National Committee VII
LDL: low-density lipoprotein
NCDs: non-communicable diseases
NHI: national health insurance
NSTEMI: non-ST segment elevation myocardial infarction
OECD: Organisation for Economic Co-operation and Development
PCI: percutaneous coronary intervention
PERKENI: Perkumpulan Endokrinologi Indonesia (Indonesian Endocrinology Association)
RR: relative risk
SCIENCE: Sardjito Intensive Cardiovascular Care
SMuRFs: standard modifiable cardiovascular risk factors
STEMI: ST segment elevation myocardial infarction
UA: unstable angina
UHC: universal health coverage
VIF: variance inflation factor
