# Supplementary material for: In-hospital mortality of patients with acute coronary syndrome (ACS) after implementation of national health insurance (NHI) in Indonesia
Source: BMC Health Serv Res. 2024 Mar 5;24:284. doi: 10.1186/s12913-024-10637-5 (PMC10916244; doi:10.1186/s12913-024-10637-5)
Supplement: Supplementary file 1 — Supplementary Material 1: Methods - The definitions of variables measured in this study [file 12913_2024_10637_MOESM1_ESM.docx]

Supplementary material

Methods

The definitions of variables measured in this study

**Definitions**

Insurance

The no insurance category included patients who paid for their treatment in the hospital out of their own pocket. The NHI is the insurance scheme provided by the central government, which started in 2014. On the other hand, the government funded insurance that was provided by the central government through a program called Asuransi Kesehatan (ASKES) for civil servants and Jaminan Kesehatan Masyarakat (JAMKESMAS), which was specifically for low-income citizens. Also, there was a regionally funded insurance programs which called Jaminan Kesehatan Daerah (JAMKESDA). All of these programs were available before the implementation of the NHI and were eliminated after the NHI began. Employee/regional government/private insurance was another insurance scheme, i.e., employer-paid insurance (Jaminan Sosial Tenaga Kerja/JAMSOSTEK), insurance paid by the regional government for low-income citizens who did not receive JAMKESMAS or who were also victims of natural disasters, and private insurance that was personally obtained by patients.

*Cardiovascular Risk Factors*

Smoking status

Patients who smoked routinely for at least 6 months before the onset of ACS were categorized as current smokers, while those who had quit smoking at least 6 months prior to onset were included in the former smoker category.

Diabetes

A medical history of diabetes was defined as either a diagnosis of DM type 2 prior to the onset of ACS or newly diagnosed DM as type 2 DM during hospitalization; new-onset diagnosis of DM with random blood glucose ≥ 200 mg/dL or fasting blood glucose ≥ 126 mg/dL, based on the guideline by World Health Organization (WHO) 2006[1]; or treatment with blood glucose-lowering drugs in the hospital. HbA1c was not a routine examination for ACS patients in this hospital and was subsequently not available in our study.

Hypertension

Hypertension was defined as a diagnosis of hypertension before the onset of ACS or treatment with a blood pressure-lowering drug or newly diagnosed hypertension with systolic blood pressure ≥ 140 mmHg and/or diastolic blood pressure ≥ 90 mmHg (Joint National Committee (JNC) VII guidelines[2])during admission at the emergency department.

Dyslipidemia

Patients were defined as having dyslipidemia if: total cholesterol ≥ 200 mg/dL, LDL-cholesterol ≥ 100 mg/dL, or triglycerides ≥ 200 mg/dL, or HDL < 40 mg/dL based on the optimal level by PERKENI 2019[3] measured at admission or patients on lipid lowering treatment.

Obesity (Asian classification)

We used the Asian classification of body mass index (BMI = kg/m^2^) provided by the Western Pacific Region of the WHO as the cutoff for obesity (BMI ≥ 25 kg/m^2^) in our study.[4]

*Diagnosis*

UA, NSTEMI, and STEMI were defined according to the ACS guideline of the Indonesian Cardiology Association 2018.[5]

*Medical treatment*

All ACS patients received dual antiplatelet therapy (acetyl-salicylic acid (ASA) and clopidogrel) according to the Indonesian ACS Guidelines 2018.[5] Anticoagulant therapy was added (enoxaparin, fondaparinux, or unfractioned heparine) according to the prior mentioned guidelines.

*Thrombolysis*

Thrombolysis or fibrinolysis was the pharmacological revascularization strategy if no invasive treatment was available, which was administered to patients with a diagnosis of STEMI. The drug of choice for this treatment in our study was either alteplase or streptokinase and was administered < 12 hours after the first symptoms according to the Indonesian ACS guideline.[5]

*Percutaneous coronary intervention (PCI)*

Percutaneous coronary intervention was defined as an invasive intervention with the goal of revascularization of the obstructed coronary artery. This strategy was performed either as primary PCI (i.e. in acute STEMI), after failed thrombolysis therapy (rescue PCI), or in NSTEMI patients during hospitalization after diagnostic coronary angiography (CAG) in case of obstructive coronary artery disease.

**References**

1. World Health Organization (WHO) Diabetes Programme. Definition and diagnosis of diabetes mellitus and intermediate hyperglycaemia : report of a WHO/IDF consultation [Internet]. 2006. Available from: http://www.who.int/diabetes/publications/diagnosis_diabetes2006/en/

2. Chobanian A V., Bakris GL, Black HR, Cushman WC, Green LA, Izzo JL, et al. Seventh report of the Joint National Committee on Prevention, Detection, Evaluation, and Treatment of High Blood Pressure. Hypertension. 2003 Dec;42(6):1206–52. doi:10.1161/01.HYP.0000107251.49515.c2

3. Perkumpulan Endokrinologi Indonesia. Pedoman Pengelolaan Dislipidemia di Indonesia 2019. PB Perkeni. 2019. p.1-7.

4. World Health Organization Western Pacific Region. The Asia Pacific perspective: Redefining obesity and its treatment. [Internet] Geneva: World Health Organization; 2000. [Cited 2023 April 22]. Available from: http://www.wpro.who.int/nutrition/documents/Redefining_obesity/en/ p.8–45.

5. Perhimpunan Dokter Spesialis Kardiovaskular Indonesia. Pedoman Tata Laksana Sindrom Koroner Akut Edisi Keempat. Jakarta : PERKI. 2018.
